# Alleviation of cisplatin-induced neuropathic pain, neuronal apoptosis, and systemic inflammation in mice by rapamycin

**DOI:** 10.3389/fnagi.2022.891593

**Published:** 2022-09-28

**Authors:** Moureq Alotaibi, Faten Al-Aqil, Faleh Alqahtani, Miteb Alanazi, Ahmed Nadeem, Sheikh F. Ahmad, Rebeca Lapresa, Metab Alharbi, Abdulrahman Alshammari, Muteb Alotaibi, Tareq Saleh, Raed Alrowis

**Affiliations:** ^1^Department of Pharmacology and Toxicology, College of Pharmacy, King Saud University, Riyadh, Saudi Arabia; ^2^Deanship of Scientific Research, King Saud University, Riyadh, Saudi Arabia; ^3^Pharmacy Services, King Saud University Medical City, Riyadh, Saudi Arabia; ^4^Institute of Functional Biology and Genomics, Consejo Superior de Investigaciones Científicas (CSIC), University of Salamanca, Salamanca, Spain; ^5^Institute of Biomedical Research of Salamanca, University Hospital of Salamanca, Consejo Superior de Investigaciones Científicas (CSIC), University of Salamanca, Salamanca, Spain; ^6^Department of Neurology, Prince Sultan Military Medical City, Riyadh, Saudi Arabia; ^7^Department of Basic Medical Sciences, Faculty of Medicine, The Hashemite University, Zarqa, Jordan; ^8^Department of Periodotics and Community Dentistry, College of Dentistry, King Saud University, Riyadh, Saudi Arabia

**Keywords:** CIPN, p21, cisplatin, rapamycin, IL-17A

## Abstract

Platinum-based chemotherapeutic treatment of cancer patients is associated with debilitating adverse effects. Several adverse effects have been well investigated, and can be managed satisfactorily, but chemotherapy-induced peripheral neuropathy (CIPN) remains poorly treated. Our primary aim in this study was to investigate the neuroprotective effect of the immunomodulatory drug rapamycin in the mitigation of cisplatin-induced neurotoxicity. Pain assays were performed *in vivo* to determine whether rapamycin would prevent or significantly decrease cisplatin-induced neurotoxicity in adult male Balb/c mice. Neuropathic pain induced by both chronic and acute exposure to cisplatin was measured by hot plate assay, cold plate assay, tail-flick test, and plantar test. Rapamycin co-treatment resulted in significant reduction in cisplatin-induced nociceptive-like symptoms. To understand the underlying mechanisms behind rapamycin-mediated neuroprotection, we investigated its effect on certain inflammatory mediators implicated in the propagation of chemotherapy-induced neurotoxicity. Interestingly, cisplatin was found to significantly increase peripheral IL-17A expression and CD8- T cells, which were remarkably reversed by the pre-treatment of mice with rapamycin. In addition, rapamycin reduced the cisplatin-induced neuronal apoptosis marked by decreased neuronal caspase-3 activity. The rapamycin neuroprotective effect was also associated with reversal of the changes in protein expression of p21^Cip1^, p53, and PUMA. Collectively, rapamycin alleviated some features of cisplatin-induced neurotoxicity in mice and can be further investigated for the treatment of cisplatin-induced peripheral neuropathy.

## Introduction

Peripheral neuropathy is an established pain disorder (Hsiao et al., 2010; St Sauver et al., 2013) that encompasses multiple types varying in their clinical presentation, severity of pain, reversibility of neuronal damage, and prognosis (Stubblefield et al., 2009). The clinical presentation of peripheral neuropathy involves a wide range of signs and symptoms that largely depend on the etiology and site of neuronal damage. The etiology of peripheral neuropathy includes genetic, autoimmune, infectious, nutritional, and metabolic imbalances. In addition, peripheral neuropathy develops as a consequence to the exposure to anticancer chemotherapeutic treatment which is often referred to as chemotherapy-induced peripheral neuropathy (CIPN). The duration, mechanism, and onset of CIPN are unpredictable, but could be linked to factors such as age, type of chemotherapy, and extent of drug exposure. Importantly, CIPN may interfere with drug dosing, patient’s compliance, and patient’s lifestyle, which, in many cases, can lead to the discontinuation of the treatment (Colvin, 2019). Although many patients with peripheral neuropathy may improve after cessation of chemotherapy, some patients’ symptoms may persist or worsen due to persistent nerve damage (Cavaletti and Marmiroli, 2010). Unfortunately, multiple treatment modalities have been suggested to manage CIPN symptoms, but no effective strategy has been yet adopted (Flatters et al., 2017).

The prototypical platinum compound, cisplatin, which is still widely used for the treatment of various malignancies, results in sensory peripheral neuropathy with a typical sock-and-glove presentation (Staff et al., 2019). Other chemotherapies that also have been shown to precipitate CIPN include microtubule poisons, bortezomib, and thalidomide (Winer et al., 2004; Jones et al., 2005; Cavaletti and Marmiroli, 2010; Richardson et al., 2012). While the ability of these drugs to cause neuropathy is established, the pathophysiology of CIPN remains largely undetermined. Some studies indicated that taxanes disrupt the neuronal microtubules and deplete axonal energy supply due to mitochondrial damage (Argyriou et al., 2012; Miltenburg and Boogerd, 2014). Other evidence has also suggested that vinca alkaloids-induced peripheral neurotoxicity occurs as a consequence to structural and functional alterations of the cytoskeleton (Argyriou et al., 2012; Miltenburg and Boogerd, 2014). On the other hand, platinum compounds toxicity was thought to be promoted through neuronal mitochondrial dysfunction and apoptosis (Kim et al., 2015). This is largely based on the ability of cisplatin to form platinum-DNA adducts in the neurons of the dorsal root ganglia (DRG) (Kim et al., 2015). Moreover, cisplatin was shown to induce alterations in calcium signaling and homeostasis, thus altering neuroexcitability of DRG neurons (Erol et al., 2016). Similar to taxanes, cisplatin can also interact with components of the cytoskeletal microtubules and result in dysfunctional neuronal transport (Tulub and Stefanov, 2001). Importantly, a large body of evidence has suggested that CIPN is mediated through an inflammatory process that is mediated by the increased production of a spectrum of pro-inflammatory cytokines and chemokines (Brandolini et al., 2019). The contribution of inflammation to the pathogenesis of neuropathy has suggested the use of anti-inflammatory compounds and immunomodulators for the treatment of CIPN (Hu et al., 2018).

Rapamycin is a well-known anticancer and immunosuppressive agent that has been found to possess neuroprotective properties (Okamoto et al., 2016; Singh et al., 2017). The anticancer mechanism of action of rapamycin is based on its ability to inhibit the mammalian target of rapamycin (mTOR), the main player in a pathway that is dysregulated in several types of malignancies (Mita et al., 2003; Bjornsti and Houghton, 2004; Majumder et al., 2004). Moreover, adding rapamycin to chemotherapeutic regimens reduced the extent of chemotherapeutic resistance in tumor cells (Guri and Hall, 2016). Furthermore, several reports in the literature revealed that rapamycin has a protective effect against many neurodegenerative processes implicated in parkinsonism both *in vitro* and *in vivo* (Pan et al., 2008; Tain et al., 2009; Malagelada et al., 2010). Other studies have shown that rapamycin treatment reduced the progression of spinal cord-induced neuropathic pain in mice (Tateda et al., 2017). Interestingly, semisynthetic analogs of rapamycin significantly promoted neurite survival and neuronal proliferation, indicating that rapamycin itself, as well as its analogs, can be used as neuroprotective agents (Ruan et al., 2008). Nevertheless, rapamycin’s effect in mitigating symptoms of CIPN has not yet been investigated in preclinical models.

In this work, we aimed to investigate the potential of a well-established immunomodulator to exert a neuroprotective effect to alleviate the neurotoxicity induced by cisplatin *in vivo* (Ta et al., 2009). We also conducted additional *in vitro* experiments to understand the underlying cellular mechanisms of cisplatin-induced neurotoxicity and rapamycin neuroprotective function (Lapresa et al., 2019). Prevention of cisplatin-induced peripheral neuropathy by rapamycin might improve cancer patients’ quality of life and increases patients’ compliance.

## Materials and methods

### Animals

Adult male Balb/c mice (8 weeks at beginning of experiments, 20–30 g) were provided by experimental animal care center at College of Pharmacy, King Saud University. All animal studies have been conducted in accordance with institutional committee of scientific research ethics of King Saud University. Mice were housed in plastic cages, exposed to a reverse 12 h light/12 h dark cycle (lights off at 7 p.m.), and were maintained in a temperature (21 ± 2^°^C), humidity (40–60%)—controlled environment. Experimental animals were allowed to acclimatize to the testing room environment for 1 week prior to the tests and experiments were performed between 9:00 a.m. and 5:00 p.m. Mice were divided into four groups: Control: received vehicle *n* = 8, Cisplatin (CIS); *n* = 8, Rapamycin (Rap); *n* = 8, Cisplatin + Rapamycin CR; *n* = 8.

### Drug administration

Cisplatin (Tocris, Cat. No. 2251) was diluted in normal saline and administered to mice intraperitoneally (i.p.). Rapamycin (Sigma-Aldrich, St. Louis, MO) was dissolved in DMSO and administered to mice subcutaneously (s.c). In the chronic treatment, mice received six doses of cisplatin (3 mg/kg) (Park et al., 2013) with or without rapamycin (3 mg/kg) every other day for 12 days, the animals were then tested 24 h after the last injection. Day 0 was set to determine the baseline of weight and other behavioral assays, and injections were given on days 1,3,5,7,9, and 11. On day 12, the final measurements were recorded. In addition to chronic cisplatin exposure, we intended to utilize a model of acute neurotoxicity within 24 h to avoid animal loss due to cisplatin-induced nephrotoxicity. For acute cisplatin exposure, mice were treated with cisplatin at a dose of 10 mg/kg (Dzagnidze et al., 2007), with or without rapamycin (3 mg/kg). Animals were tested 4 and 24 h after the injection. In both models, rapamycin was s.c. injected on the same day 3 h prior to the cisplatin injection and control mice were treated with normal saline and tested at all the corresponding time points. We start the study with 12 animals per group. Few mice per group (except control mice) were lost, due to either infections following repeated injections or cisplatin-induced nephrotoxicity. Weight loss was observed in cisplatin-injected mice as well as the combination (average weight was ∼20 g), whereas the average body weight reached 30 g in control mice. Our exclusion criteria were based on body weight and locomotor activity (LA).

### Behavioral assays

#### Locomotor activity test

LA was measured as described previously with only minor modifications (York et al., 2013). Briefly, LA was measured with a Supermex apparatus (Muromachi Kikai, Tokyo, Japan) and a sensor monitor that was mounted above the chamber. The open field arena of the chamber was 25 cm × 45 cm × 20 cm. Each mouse was housed from its home cage to habituate to the testing room environment. The activity of the mice was measured by using activity monitoring software (AMS). Then put the mouse in the testing chamber and started the 10 min test session. Exploratory activity count was summed automatically in the test session.

#### Hot plate test

Animals were placed on a hot-plate apparatus (Ugo Basile 35100, United Kingdom). In order to measure thermal pain, temperature of the hot plate surface was set to 52 ^°^C. The reaction latency as well as the time between when the animals were placed and the onset of paw-licking or jumping behaviors were measured in seconds (s). To minimize tissue damage, a cut-off time of 40 s was adopted.

#### Cold plate test

The animals were placed in a stainless-steel box (12 cm × 20 cm × 10 cm) with a cold plate as floor with temperature kept constant at 4^°^C ± 1^°^C. Licking of the hind paw due to pain was observed and the time (seconds) of the first sign was recorded. The cut-off time of the latency of paw lifting or licking was set at 60 s.

#### Tail-flick test

Thermal (radiant heat) sensation of mice tail part was assessed by observation of tail flick response as per D’Aemour and Smith method with minor modifications (Hargreaves et al., 1988). The one-centimeter distance from the tail terminal region was located on the slit of the plantar device. Thermal sensitivity of mice tail was recorded as tail withdrawal latency. Brisk withdrawal of tail from the plantar device was considered as a progression of neuropathic pain. Cut-off stimuli, i.e., 15 s was maintained to avoid potential tissue damage. Mice were tested after 24 h of treatment injections for the drug-response evaluation.

#### Plantar test

The pain sensitivity to thermal heat was tested according to the Hargreaves procedure using the plantar test (Hargreaves et al., 1988). Mice were placed in a plexiglass enclosure with transparent glass floor. Infrared beam was passed as a heat source at the hind paw surface. The latency to the first sign of paw licking or withdrawal response to avoid heat pain was taken as an index of pain threshold. The withdrawal latency was averaged from at least three trials separated by a 5-min interval and the cut- off was set at 20 s to avoid tissue damage.

### Assessment of the pro-inflammatory cytokine, IL-17A in CD4^+^ and CD8^+^ T cells by flow cytometry

Flow cytometry analysis was performed to evaluate intracellular IL-17A levels in CD4^+^ and CD8^+^ T cells from blood as described before (Ahmad et al., 2018; Nadeem et al., 2020). Whole blood (100 μl) was pipetted directly into a 12 × 75 mm fluorescence activated cell sorting tube containing 5 μl of monoclonal antibodies against the CD4^+^ and CD8^+^ T cell surface antigen (BioLegend; San Diego, USA), followed by incubation at room temperature in the dark for 10 min. Red blood cells (RBCs) were lysed with 2 ml of 1 × lysing solution (BD Biosciences, USA) for 10 min. After centrifugation at 300 × *g* for 5 min, the supernatant was discarded, and 1 × permeabilizing solution (3 ml, BioLegend; San Diego, USA) was added to the pellet, followed by incubation for 10 min at room temperature in the dark. After washing with 3 ml of wash buffer, cytokine specific antibody (5 μl) for detection of intracellular expression of IL-17A (BioLegend; San Diego, USA) was added to the cells, followed by incubation for 30 min at room temperature in the dark. After one final wash, 10,000 cells were acquired for flow cytometric analysis and were analyzed using CXP software (Beckman Coulter, USA) as detailed previously (Ahmad et al., 2018; Nadeem et al., 2020).

### Neuronal cultures

Primary cultures of mice cortical neurons were prepared from fetal C57BL/6 mice of 14.5 days of gestation, seeded at 1.8⋅10^5^ cells/cm^2^ in 24-well plastic plates coated with poly-D-lysine (10 μg/ml) and incubated in Neurobasal-A medium (Life Technologies) supplemented with 5.5 mM of glucose, 0.25 mM of pyruvate, 2 mM of glutamine and 2% B27 supplement (Life Technologies). Cells were incubated at 37^°^C in a humidified 5% CO_2_-containing atmosphere. At 72 h after plating, medium was replaced. Cells were used at day 8–9 (Lapresa et al., 2019).

### Neurotoxicity assay

Cortical neurons were incubated in culture medium containing 10 μM Cisplatin ± 100 nM Rapamycin for 24 h. Control neurons were incubated in the absence of treatments. The toxicity of cisplatin-rapamycin co-treatment was evaluated by the activity of caspase-3 (Fluorimetric Caspase-3 Assay Kit, Sigma-Aldrich), according to the manufacturer’s instructions. This assay is based on the hydrolysis of the peptide substrate Ac-DEVD-AMC (acetyl-Asp-Glu-Val-Asp-7-amino-4-methylcoumarin) by caspase-3, which results in the release of fluorescent 7-amino-4-methylcoumarin (AMC). Caspase-3 activity was determined as AMC release rate extrapolating the slopes to those obtained from an AMC standard curve. Results are expressed as mean activity ± SEM from 5 different neuronal cultures.

### Western blot analysis

Neurons were lysed in RIPA buffer (2% sodium dodecylsulphate, 2 mM EDTA, 2 mM EGTA and 50 mM Tris pH 7.5), supplemented with protease and phosphatase inhibitor cocktail (100 μM phenylmethylsulfonyl fluoride, 50 μg/ml antipapain, 50 μg/ml pepstatin, 50 μg/ml amastatin, 50 μg/ml leupeptin, 50 μg/ml bestatin, 1 mM o-vanadate, 50 mM NaF, and 50 μg/ml soybean trypsin inhibitor), stored on ice for 30 min and boiled for 5 min. Protein concentrations were determined with the BCA (bicinchoninic acid) method, using bovine serum albumin as a standard (BCA Protein Assay kit, Thermo Fisher Scientific). Neuronal extracts were subjected to SDS-polyacrylamide gel (MiniProtean; Bio-Rad). The antibodies used include anti-p53 (1:2,000; 2,524, Cell Signaling Technology), anti-p21^Cip1^ (1:500; 556,431, Becton Dickinson Biosciences); anti-PUMA (1:1,000; ab54288, Abcam, Cambridge, UK); and anti-GAPDH (1:40,000; Ambion, Cambridge, UK) overnight at 4^°^C. GAPDH was used as the loading control. After incubation with horseradish peroxidase-conjugated goat anti-rabbit IgG (Pierce, Thermo Scientific) or goat anti-mouse IgG (Bio-Rad), membranes were incubated with the enhanced chemiluminescence SuperSignal West Femto (Pierce) for 5 min Pierce ECL Plus Western Blotting Substrate (Thermo Fisher Scientific) for 5 min, before exposure to Kodak XAR-5 film, and the autoradiograms were scanned (22). Band intensities were quantified using ImageJ1.48v software.

### Statistical analysis

Data were statistically analyzed using two-way analysis of variance (ANOVA) followed by Tukey’s *post hoc* using graph pad 5.0 prism software (GraphPad Software). The *p*-value was considered as statistically significant if *p < 0.05, ^**^p < 0.01, ^***^p < 0.001, and ^****^p < 0.0001 between treatment groups. All the data are represented by Means ± SEM.

## Results

### Chronic administration of rapamycin reversed cisplatin-induced thermal hypersensitivity in mice

**FIGURE 1:**
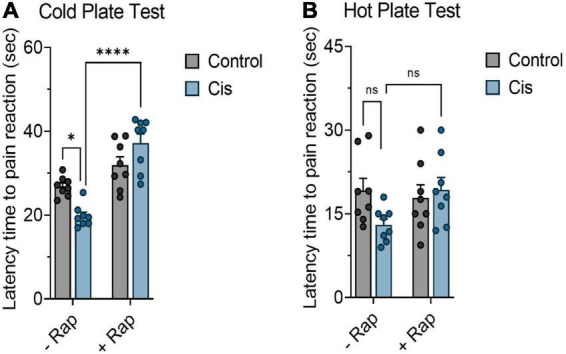
Effect of rapamycin on thermal hypersensitivity induced by chronic exposure to cisplatin. Balb/c mice were treated with cisplatin 3 mg/kg (i.p.) with or without rapamycin 3 mg/kg (s.c.) every other day for 12 days. **(A)** Impact of rapamycin on cisplatin using cold plate test. **(B)** Impact of rapamycin on cisplatin using hot plate test. *p < 0.05, ^**^p < 0.01, ^***^p < 0.001, and ^****^p < 0.0001 indicates statistical significance between treatment groups (two-way ANOVA followed by Tukey’s *post-hoc* test; *n* = 8 mice). ns, non-significant.

In order to assess the effect of rapamycin on cisplatin-induced neuropathic pain, mice were treated with cisplatin (3 mg/kg) with or without rapamycin (3 mg/kg) as indicated in section “Materials and methods.” Mice were then tested for pain sensitivity using cold plate test, hot plate test, tail-flick test, and plantar test. Data from the cold plate test showed that the latency time of animals in the cisplatin-treated group was remarkably reduced in comparison with the vehicle-treated group. However, administration of 3 mg/kg (s.c.) of rapamycin along with cisplatin significantly increased the reaction time as compared to the cisplatin-treated group (Figure 1A). Consistent with this observation, hot plate results demonstrated that pre-treatment with rapamycin has prolonged the latency time in comparison to cisplatin only group; indicating that mice reaction to pain source has been attenuated (Figure 1B). In addition, we measured body weight and rectal temperature in all mice groups. Although cisplatin-treated group showed weight loss, the co-treatment with rapamycin did not have a major effect compared to the cisplatin only group (data not shown). These results indicate that chronic treatment with rapamycin in combination with cisplatin reduced thermal hypersensitivity with no major effects on weight and LA.

### Rapamycin suppresses pain induced by a single, high dose of cisplatin

**FIGURE 2:**
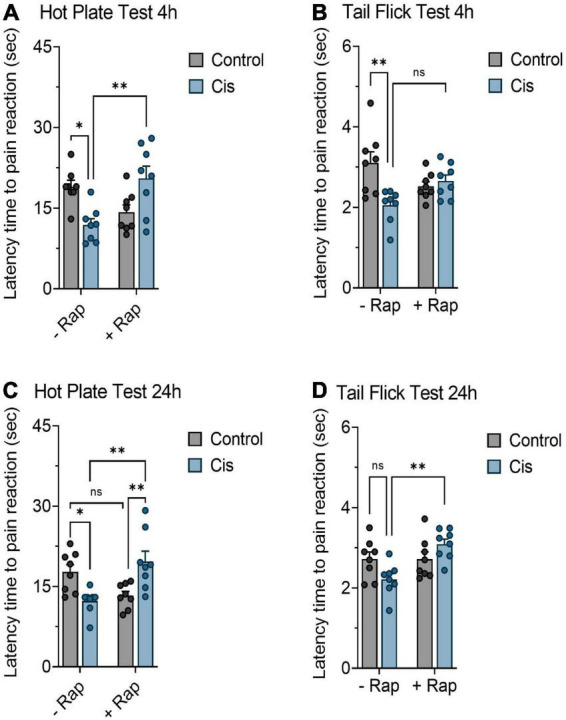
Effect of rapamycin on thermal hypersensitivity induced by acute exposure to cisplatin. Balb/c mice were treated with a single dose cisplatin 10 mg/kg (i.p.) with or without rapamycin 3 mg/kg (s.c.) for 24 h. **(A,C)** Impact of rapamycin on cisplatin using hot plate test 4 and 24 h post-treatment, respectively. **(B,D)** Impact of rapamycin on cisplatin using tail flick test 4 and 24 h post-treatment, respectively. *p < 0.05, ^**^p < 0.01, ^***^p < 0.001, and ^****^p < 0.0001 indicates statistical significance between treatment groups (two-way ANOVA followed by Tukey’s *post-hoc* test; *n* = 8 mice).

Since the neurotoxic effects of chronic platinum-based compounds have been documented (Lehky et al., 2004; Park et al., 2009), we next wanted to measure the ability of rapamycin to attenuate thermal hypersensitivity produced by a single dose of cisplatin in mice. Mice were injected with a single high dose of cisplatin (10 mg/kg) with or without rapamycin (3 mg/kg) and pain measurements were recorded at 4 and 24 h post-treatment. Unlike the hot plate test, which showed that rapamycin was successful in moderately reversing cisplatin-induced thermal hypersensitivity (Figure 2A), we have not observed significant changes in response using the tail flick test at 4 h post-treatment (Figure 2B). However, the withdrawal responses were significantly increased in the rapamycin plus cisplatin-receiving group in comparison with the cisplatin-treated group after 24 h of treatment, as shown in Figures 2C,D. Similarly, LA and motor coordination in mice, body weight, and rectal temperature were measured throughout the monitoring period and no significant differences were found among groups (data not shown). These data indicate that rapamycin can alleviate components of high-dose, cisplatin-induced nociceptive-like symptoms in mice.

### Effect of cisplatin and rapamycin on systemic inflammation

**FIGURE 3:**
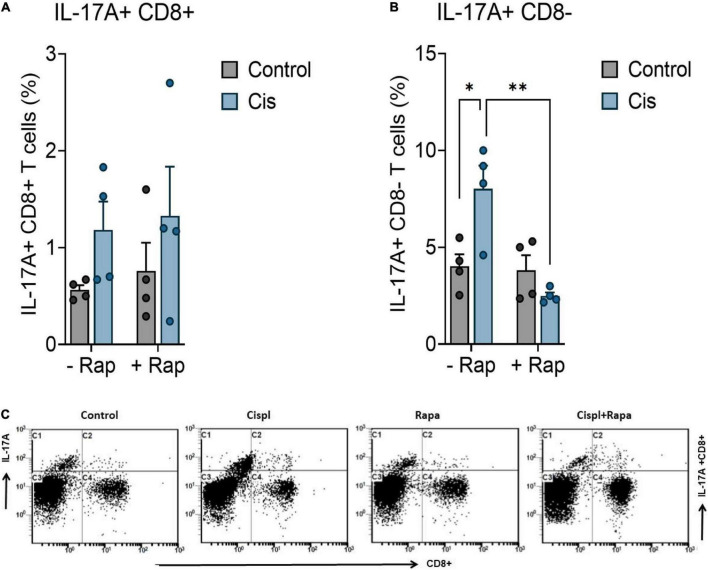
Rapamycin reduces the systemic inflammation. Balb/c mice were treated with a single dose cisplatin 3 mg/kg (i.p.) with or without rapamycin 3 mg/kg (s.c.) for 12 days. **(A)** Impact of rapamycin on cisplatin-induced effect on IL-17A expression in CD8 + T cells. **(B)** Impact of rapamycin on cisplatin-induced effect on IL-17A expression in CD8- T cells. IL-17A expression was evaluated in the blood by flow cytometry. *p < 0.05, ^**^p < 0.01, ^***^p < 0.001, and ^****^p < 0.0001 indicates statistical significance between treatment groups. (two-way ANOVA followed by Tukey’s *post-hoc* test; *n* = 4 mice). **(C)** Representative flow cytometry plots for evaluation of intracellular IL-17A levels in CD4 + and CD8 + T cells from blood in treated mice. ns, non-significant.

Cisplatin has been reported to induce peripheral neuropathy through activation neuroinflammatory pathways (Starobova et al., 2020). Since rapamycin is known to have inflammation modulatory effects, we hypothesized that its effects against neuropathy is mediated by the attenuation of cisplatin-mediated neuroinflammation. Accordingly, we examined whether cisplatin administration caused systemic inflammation and whether that was attenuated by rapamycin treatment. For this, we measured IL-17A expression in CD4 + and C8 + T cells. IL-17A was selected because of its previously described role in mediating neuroinflammation (Nadeem et al., 2017, 2018, 2019). Cisplatin administration did not cause significant elevation of IL-17A expression in CD8 + T cells (Figure 3A)/CD4 + T cells (data not shown), however it caused significant increase in overall expression of IL-17A in CD8-T cells (Figure 3B). Furthermore, treatment with rapamycin caused significant attenuation in cisplatin-induced expression of IL-17A expression in CD8- T cells (Figure 3C).

### Rapamycin reduces neurotoxicity induced by cisplatin in neuronal culture

**FIGURE 4:**
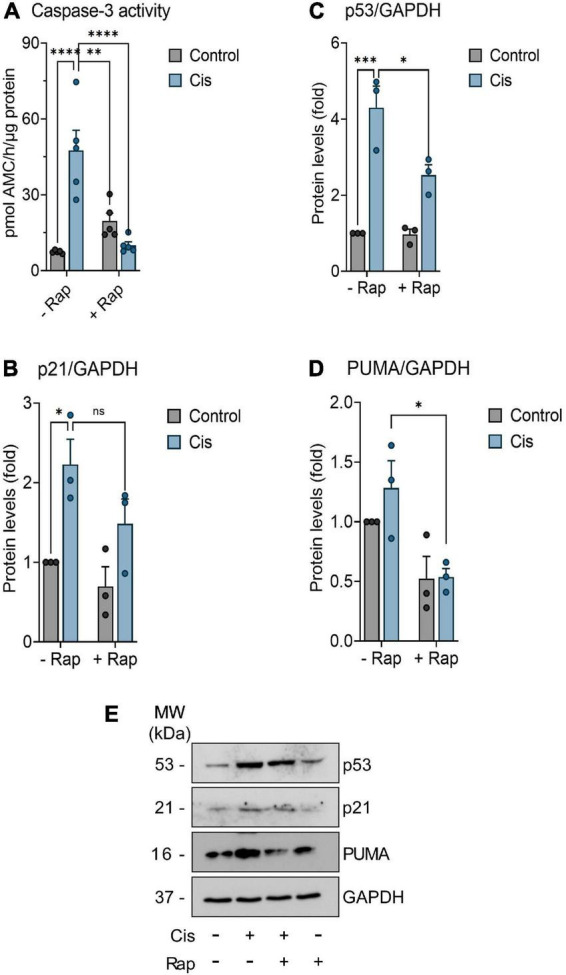
Assessment of senescence and apoptosis in neurons. Neurons were further incubated in culture medium containing or not (control) 10 μM Cisplatin (Cis), either in the absence or presence of 100 nM Rapamycin (Rap) for 24 h. When indicated, neurons were pre-treated with 100 nM Rapamycin for 3 (-3 h) hours before Cisplatin exposure. **(A)** Fluorometric detection of caspase-3 activity was performed as an index of neuronal apoptosis (*n* = 5). **(B–D)** Neurons were treated with indicated treatment as mention previously for 24 h. Thereafter, p53, p21, and PUMA proteins expression levels were determined by Western blot analysis. Values are expressed as mean ± SEM. *p < 0.05, **p < 0.01, ***p < 0.001, and ****p < 0.0001 indicates statistical significance between treatment groups (two-way ANOVA followed by Tukey’s *post-hoc* test; *n* = 3). **(E)** Representative western blot showing protein level of p53, p21, and PUMA in neuronal cultures. ns, non-significant.

In order to study the potential neuroprotective effect of rapamycin upon cisplatin-induced neurotoxicity, we extended our studies to an *in vitro* model. For that, we used primary cultures of mice cortical neurons prepared from fetal C57BL/6 mice (Lapresa et al., 2019). Primary culture neurons were subjected to treatment regimens as outlined in the Methods. To investigate cisplatin-induced neurotoxicity, we measured caspase-3 activity, an established indicator of neuronal apoptosis (Gill and Windebank, 1998). Determination of caspase-3 activity revealed that 10 μM cisplatin have induced neurotoxicity within 24 h post-treatment, whereas exposure to 100 nM rapamycin did not result in a significant change in caspase-3 activity relative to control (Figure 4A). Interestingly, 3 h pre-treatment with rapamycin reduced cisplatin-induced neuronal apoptosis, as revealed by the decrease in caspase-3 activity 24 h post-treatment. In addition to apoptosis, several reports have suggested a role for cellular senescence in the pathogenesis of neurodegeneration (Bhat et al., 2012; Birger et al., 2019; Nicaise et al., 2019; PerezGrovas-Saltijeral et al., 2020). Thus, the expression of further markers of apoptosis and senescence were assessed. Protein levels of PUMA, p53, and p21^Cip1^ have been measured using immunoblotting and have been reduced by rapamycin treatment, suggesting that the neuroprotective role of rapamycin might be through the suppression of apoptosis and senescence (Figures 4B–E).

## Discussion

The development of peripheral neuropathy is a treatment-limiting adverse effect of cancer therapy. CIPN may impinge on optimal drug dosing, patient’s compliance to treatment, and patient’s lifestyle, which collectively can lead to a premature discontinuation of chemotherapeutic treatment. The current major challenge in CIPN research include identifying effective pharmacological therapy that will both mitigate symptoms and allow for the completion of anticancer treatment. Recent evidence have shown that different compounds have neuroprotective properties and demonstrated promising effects in reducing CIPN in preclinical models (Galley et al., 2017; Kyte et al., 2018). However, these studies do not confirm whether these agents would prevent tumor progression or interfere with the antitumor potential of chemotherapy. For example, nicotine, which was shown to be effective in alleviating CIPN in mice, has already been found to be a tumor promotor (Sun et al., 2017; Wang et al., 2017; Zhang et al., 2017). Therefore, it is advantageous to investigate neuroprotective agents that not only lack a pro-tumorigenic potential, but also possess beneficial anticancer properties. Additionally, understanding the exact underlying mechanisms that contribute to CIPN is essential. Several processes are implicated in the pathogenesis of CIPN, including oxidative stress, dorsal root neuronal apoptosis, altered calcium homeostasis, mitochondrial dysfunction, and axonal degeneration leading to disturbed neuronal transport (Colvin, 2019). Moreover, the ability of chemotherapeutic agents to cause neurotoxicity *via* inflammatory processes has recently emerged as a fundamental element in the development of CIPN (Fumagalli et al., 2021). Subsequently, the investigation of immunomodulatory drugs for CIPN is now of great interest. Finally, despite that CIPN is more frequently reported in female human patients receiving cancer chemotherapy, preclinical evidence show no gender contribution to development of CIPN in murine models (Carpenter et al., 2021). However, our data would benefit from confirmation of rapamycin’s neuroprotective effect in female mice.

Our work provides data on the utility of rapamycin, an established immunomodulatory agent with anticancer effects, to propagate neuroprotection in a model of cisplatin-induced peripheral neuropathy. While cisplatin clearly induced neuronal damage accompanied by the development of thermal hypersensitivity, pre-treatment with rapamycin protected animals from exacerbated pain in cisplatin-treated groups. Response of rodents to thermal stimulators has been previously demonstrated in several studies based on thermal tests (hot plate, cold plate, plantar test, and tail flick test) (Deuis et al., 2017). Rapamycin prolonged the latency time by which animals reacted to the thermal inducers in the cisplatin-treated group as shown in Figure 1. These data are in agreement with previous studies showing that rapamycin has neuroprotective properties in other models of neuronal damage (Asante et al., 2010; Wang et al., 2016). In addition, rapamycin still exerts its effect (Figure 2) even when cisplatin was given acutely at higher dose (10 mg/kg), indicating that rapamycin is a potent neuroprotective agent. Several reports have indicated that some anticancer agents including cisplatin induce cell death in neuronal cultured from mice (Üstün et al., 2018). Thus, we intended to expand our experiments to include an *ex vivo* model. Consistent with our animal experiments, rapamycin has remarkably reduced apoptosis after exposure to cisplatin in a time-dependent manner (Figure 3). Taken together, rapamycin obviously attenuated the cisplatin-induced neurotoxicity both *in vivo* and *ex vivo*.

The neuroprotective effect of rapamycin in in this work can be attributed to several mechanisms. Recent studies showed that the neuroprotective effect of rapamycin is dependent on mTOR pathway blockade (Wang et al., 2016), whereas others pointed toward a reduction in the secretion of a spectrum of pro-inflammatory cytokines (Duan et al., 2018). Here, we sought to investigate whether the neuroprotective action of rapamycin is associated with modulation of systemic inflammation. Interestingly, we found that cisplatin significantly increased overall systemic inflammation, i.e., IL-17A expression in CD8 + T cells, whereas pre-treatment of mice with rapamycin remarkably reversed the cisplatin-induced effects on IL-17A expression (Figure 3). Earlier studies have shown an important role of IL-17A in causation of chemotherapy-induced neuropathic pain (Yao et al., 2016; Luo et al., 2019). Furthermore, reports have indicated that mTOR pathway is involved in mediating IL-17A production (Gulen et al., 2010; Nagai et al., 2013; Raïch-Regué et al., 2015; Chen et al., 2020), which is considered as a neurotoxic cytokine (Li et al., 2007; Kebir et al., 2007; Bǎlaşa et al., 2013; Debnath and Berk, 2014). IL-17A is a pro-inflammatory cytokine that is involved in neuroinflammatory and neuropsychiatric disorders such as autism, depression, and multiple sclerosis. It has been shown to be predominantly expressed by T cells such as CD8 + /CD4 + T cells. In the current study, IL-17A + immunostaining in CD8 + T/CD4 + T cells was not significantly different among different groups. However, IL-17A + immunostaining in immune cells other than CD8 + T cells was found be increased in cisplatin group which was significantly downregulated by rapamycin treatment. This observation suggests that rapamycin likely reduced expression of IL-17A in immune cells other than CD8 + T cells. Immune cells other than CD8 + /CD4 + T cells which express and secrete IL-17A include gamma delta T cells, NK cells, B cells, and granulocytes. Rapamycin-mediated reduction in IL-17A expression may occur in these immune cells. Future studies are required to ascertain which specific immune cells are affected by rapamycin treatment. Overall, our data point toward the possibility of IL-17A involvement in chemotherapy-induced neuropathic pain.

In addition to its immunomodulatory effects, rapamycin appears to decrease neuronal cell death. In our work, rapamycin decreased the expression of markers of apoptosis and senescence in response to cisplatin (Figure 4). Caspase-3 activity and the levels of PUMA, p53 and p21^Cip1^ were found to be elevated when cells were exposed to cisplatin alone indicating genotoxic stress. In line with our findings, a recent paper by Hui et al. (2022) indicated that caspase 3 induction is necessary to for cisplatin-induced apoptosis in a neurotoxicity model and is considered a reliable marker for neuronal cell death (Hui et al., 2022). Several other studies unveiled the significance of the pro-apoptotic gene (PUMA) in neurodegeneration (Maor-Nof et al., 2016; Simon et al., 2016). Thus, it is of value to assess whether PUMA induction and caspase 3 activation are linked to cisplatin-induced neuroapoptosis. However, we encourage that future studies of cisplatin-induced neurotoxicity include cell viability assays along with more biomarkers of apoptosis such as annexin V staining and TUNEL assay. Recent evidence has suggested that cellular senescence plays a significant role in the development of CIPN. The first report by Acklin et al. (2020) showed that primary cultured DRG neurons isolated from adult C57BL/6 mice exposed to cisplatin *ex vivo* have upregulated senescence-associated-β-gal staining, a hallmark of senescence. Moreover, cisplatin treatment resulted in increased cytoplasmic staining of both p16^INK4a^, a canonical marker of senescence, and HMGB1 of primary DRG neurons (Acklin et al., 2020). In peripheral neuropathy, primary DRG neuronal culture or DRG organotypic cultures are often used to perform *in vitro* experiments due to the high susceptibility of DRG neurons against cisplatin toxicity (Haberberger et al., 2020). However, like every model, these also have a downside, which is the heterogeneous neuronal population, complicating in some cases the drawing of conclusions. Besides, the neurotoxic effects of cisplatin-chemotherapy are not limited to the periphery, but also severely affect CNS neurons, giving rise to the term “chemobrain” (Chiu et al., 2017). Furthermore, the p53-dependent apoptotic neuronal death is a mechanism shared among neurons, regardless of whether they are in the central or peripheral nervous system (Krukowski et al., 2015; Chiu et al., 2017; Maj et al., 2017). Therefore, we chose to perform our experiments on the less heterogeneous primary cortical neuronal cell culture as the most suitable model to test our hypothesis, which has been also used to study chemotherapy induced peripheral neuropathy (Lehmann et al., 2020). These observations of senescence were extended to an *in vivo* CIPN model by Calls et al. (2021) who indicated that, in cisplatin treated BALB/c mice, which developed CIPN in 14 weeks of treatment, DRGs exhibited senescence-like features including: positive SA-β-gal staining, large, fused mitochondria, and accumulating lipofuscin granules (Calls et al., 2021). Moreover, single-cell RNAseq of DRG’s sensory neurons revealed significant upregulation of *Cdkn1a* which encodes for p21^Cip1^ in agreement with our findings (Calls et al., 2021). Consequently, the removal of cisplatin-induced senescent cells reversed, to an extent, behavioral features of CIPN. While these cells were eliminated successfully by senolytic drugs (agents that selectively kill senescent cells), the use of senomorphics (agents that modulate the inflammatory phase of senescence) is encouraged (Carpenter et al., 2021). Accordingly, in our study, rapamycin can be hypothesized to have exerted its neuroprotective effect through its senomorphic potential, which was marked by the reduction of the senescence marker p21^Cip1^ and the inflammatory mediator IL-17A (Laberge et al., 2015; Xu et al., 2021).

## Conclusion

In conclusion, rapamycin showed effectiveness against cisplatin-induced peripheral neuropathy. Our behavioral analysis revealed the ability of rapamycin to reduce hypersensitivity to thermal inducers even at high doses. Also, rapamycin significantly decreased caspase activity, a marker of apoptotic cell death, in cisplatin-treated neurons. Furthermore, the increase of pro-inflammatory cytokine expression, IL-17A, was seen to be associated with cisplatin use, and its expression was reversed by pre-treatment with rapamycin. Further studies are needed to elaborate more about the involvement of inflammatory signals in chemotherapy-induced neuropathic pain. Collectively, addition of rapamycin with neuropathic pain-inducing anticancer agents might be beneficial to cancer patients and could improve cancer therapy outcomes.

## Data availability statement

The raw data supporting the conclusions of this article will be made available by the authors, without undue reservation.

## Ethics statement

This animal study was reviewed and approved by Dr. Musaad Ali Alshammari The Chairman of the Research Ethics Sub-Committee on Living Creatures.

## Author contributions

FA-A was the fellow while MoA was the principal investigator of the project. MoA, MuA (physician), and TS developed the idea behind the project. MiA, MeA, and AA partially did the experimental design and data analysis. FA was an expert in neuroscience research and conceptually contributed to parts related to neuroscience. AN and SA performed the studies relating to neuroinflammation. RL was involved heavily in experiments relating to apoptosis and senescence. MoA and FA-A contributed to writing, performing experiments, data collection, and analysis. All authors contributed to the article and approved the submitted version.
